# Can we predict overall survival using machine learning algorithms at 3-months for brain metastases from non-small cell lung cancer after gamma knife radiosurgery?

**DOI:** 10.1097/MD.0000000000037084

**Published:** 2023-02-02

**Authors:** Hyeong Cheol Moon, Byung Jun Min, Young Seok Park

**Affiliations:** a Department of Neurosurgery, Gamma Knife Icon Center, Chungbuk National University Hospital, Cheongju, Republic of Korea; b Department of Radiation Oncology, Chungbuk National University Hospital, Cheongju, Republic of Korea; c Department of Neurosurgery, Chungbuk National University, Cheongju, Republic of Korea.

**Keywords:** gamma knife radiosurgery, machine learning algorithms, non-small cell lung cancer

## Abstract

Gamma knife radiosurgery (GRKS) is widely used for patients with brain metastases; however, predictions of overall survival (OS) within 3-months post-GKRS remain imprecise. Specifically, more than 10% of non-small cell lung cancer (NSCLC) patients died within 8 weeks of post-GKRS, indicating potential overtreatment. This study aims to predict OS within 3-months post-GKRS using machine learning algorithms, and to identify prognostic features in NSCLC patients. We selected 120 NSCLC patients who underwent GKRS at Chungbuk National University Hospital. They were randomly assigned to training group (n = 80) and testing group (n = 40) with 14 features considered. We used 3 machine learning (ML) algorithms (Decision tree, Random forest, and Boosted tree classifier) to predict OS within 3-months for NSCLC patients. And we extracted important features and permutation features. Data validation was verified by physician and medical physicist. The accuracy of the ML algorithms for predicting OS within 3-months was 77.5% for the decision tree, 72.5% for the random forest, and 70% for the boosted tree classifier. The important features commonly showed age, receiving chemotherapy, and pretreatment each algorithm. Additionally, the permutation features commonly showed tumor volume (>10 cc) and age as critical factors each algorithm. The decision tree algorithm exhibited the highest accuracy. Analysis of the decision tree visualized data revealed that patients aged (>71 years) with tumor volume (>10 cc) were increased risk of mortality within 3-months. The findings suggest that ML algorithms can effectively predict OS within 3-months and identify crucial features in NSCLC patients. For NSCLC patients with poor prognoses, old age, and large tumor volumes, GKRS may not be a desirable treatment.

## 1. Introduction

Brain metastases (BM) are commonly considered intracranial tumors that pose a significant treatment challenge, and patient symptoms need to be controlled.^[1]^ The incidence of BM has been increased, which could be attributed to treatment methods such as traditionally surgery, radiotherapy, and chemotherapy that achieve longer overall survival (OS). Untreated BM patients have median OS around 2 months with central nervous system involvement.^[2]^ Advanced in local treatments such as gamma knife radiosurgery (GKRS) has been growing popular for BM patients.^[3–5]^ GKRS provides better local control, fewer complications than whole brain radiation therapy, and is similarly effective as surgery.^[6]^ GKRS remains difficult to local control for large BM (diameter > 3 cm, volume > 10 cc) due to radiation toxicity.^[7–10]^ Recently, a new generation Gamma Knife ICON with mask fixation has shown potential for hypo-fractionated treatments for large BM. However, despite advanced treatments, the median OS of BM patients is approximately 1 year.^[11,12]^

Lung cancer is popular malignancy worldwide. There are 2 main forms of lung cancer: non-small cell lung cancer (NSCLC) and small cell lung cancer. The NSCLC is classified into 3 main types (adenocarcinoma, squamous cell carcinoma, and large cell) by World Health Organization.^[13,14]^ Intracranial BM occurs in about 30% to 40% of NSCLC patients.^[15–17]^ GKRS has been proven to be a low-complication and effective treatment method for BM in NSCLC patients.^[18,19]^ The median OS for BM in NSCLC patients is approximately 1 year.^[18,20]^ Patients with NSCLC who develop BM have a limited survival prognosis. Typically, about 10% of BM patients die within 2-months.^[21,22]^ In comparison, our hospital data show that 10% of NSCLC patients with BM died within 3-months. The appropriateness of administering GKRS to NSCLC patients with BM who face a short overall survival is debatable. Furthermore, Korean health insurance policies permit GKRS to be performed every 3 months for BM treatment.

Many clinical decision are based on consensus agreements and recommendations, especially when the evidence is not fully sufficient.^[23]^ Learning from information with simple structures makes it easy to model observations and draw conclusions.^[24]^ Machine learning (ML) is a subset of artificial intelligence, enabling computer systems to learn from experience without explicit programming code. Among of ML algorithms, tree-based ML is simple to understand and to interpret. Trees can be visualized. A decision tree is a formalism for expressing decisions through nodes linked to subtrees and labeled with specific outcomes.^[25,26]^ Decision trees provide high classification accuracy and a simple representation of information in medical decision-making. However, small data sets could lead to overfitting and not guarantee optimal trees. Another method, the random forest, is an ensemble algorithm that considers results of similar or different classifications. Although random forest takes longer than a single decision tree and has slower training, it is biased with categorical variables. The boosted decision tree algorithm can minimize errors due to its extensive coverage of variable relationships within the data, thereby improving accuracy.^[27]^

In this study, we investigated the prediction of OS within 3-months after GKRS in NSCLC patients using tree models (decision tree, random forest, boost tree classifier). We identified important features and permutation features for the prediction of OS within 3-months.

## 2. Methods

### 2.1. Study protocol and patient characteristics

The study protocol retrospectively reviewed the medical records of patients treated with GKRS of BM of NSCLC, between December 2017 and July 2020 at Chungbuk National University Hospital. The study protocol was approved by the Institutional Review Board (2021-02-008-004), and it adhered to the guideline of Declaration of Helsinki (1975) for human research. The requirement of informed consent was waived on existing clinical data. We excluded the patients who were lost to follow-up before the 3-months.

**Figure 1.**
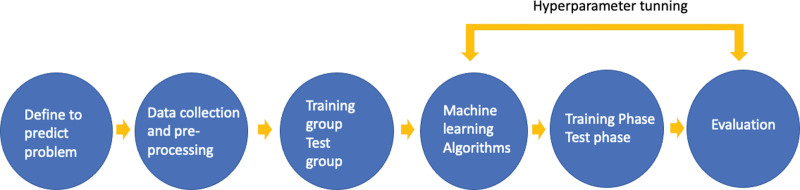
Schematic of the machine learning study design.

A total 120 patients (76 males and 44 females; age range 49 to 86 years old; median age, 71.5 years old) who were previously diagnosed with BM in NSCLC patients and were enrolled in this study. General characteristics including age, sex, karnofsky performance scale (KPS) score,^[28]^ recursive partitioning analysis class,^[29]^ pathological diagnosis, pretreatment, receiving chemotherapy, the number of lesions, tumor volume, the number of fractions, prescription dose, and OS were summarized in Table 1. The study design was shown in Figure 1.

**Table 1:** Patient demographics for brain metastases of non-small cell lung cancer.

Characteristics	Value
Number of patients	120
Age (yr)	
Median (range)	71.5 (49–86)
Sex	
Male (%)	76 (63%)
Female (%)	44 (37%)
KPS score	
100	2 (1.6%)
90	8 (6.7%)
80	84 (70%)
70	26 (21.7%)
RPA class	
I	39 (32.5%)
II	75 (62.5%)
III	6 (5%)
Pathologic diagnosis	
Adenocarcinoma	99 (82.5%)
Squamous cell carcinoma	19 (15.8%)
Large cell carcinoma	0 (0%)
Others	2 (1.7%)
Pretreatment	
Cyst aspiration	4
Whole brain radiotherapy	2
Fixation methods	
Frame	27
Mask	93
Receiving chemotherapy	18
The number of lesions	
Median (range)	2 (1–11)
1–3	88 (73.3%)
4–6	20 (16.7%)
7–10	12 (10%)
The number of tumor volume (mm^3^)	
<5 mm^3^	331
<10 mm^3^	20
>10 mm^3^	9
The number of fractions	
Single-session median (range)	98 (1)
Hypo-fractionated median (range)	22 (3–6)
The number of prescription dose (50% margin)	
<10 Gy	67
<20 Gy	63
>20 Gy	230
Overall survival (mo) median (range)	6 (1–24)

KPS = Karnofsky Performance Score, RPA = recursive partitioning analysis.

### 2.2. Treatment planning for GKRS

All patients underwent GKRS using the Leksell Gamma Knife ICON (Elekta AB, Stockholm, Sweden). All patients underwent 3 Tesla magnetic resonance imaging (Philips Achieva, Netherlands) with 32-channel head coil. The scanning sequences included T1-weighted, T1-weighted with gadolinium enhancement, and T2-weighted images. All magnetic resonance imaging images were registered with Leksell Gamma Plan (LGP, Version 11.1.1), and any images with motion artifacts were excluded. The tumor volumes were calculated by LGP without margin. Generally, the prescription dose was determined by the recommendations from the dose escalation trial Radiation Therapy Oncology Group 90 to 05,^[30]^ considering individual patient conditions. For large BM (>10 cc), fractionated GRKS was selected based on the linear quadratic model.^[31,32]^ The GKRS planning was determined through a consensus between the neurosurgeon and the medical physicist.

### 2.3. Medical data labeling

To facilitate medical data labeling, feature extraction was performed on the general characteristics within the electronic medical record (EMR). Missing data in the EMR is a common challenge in ML tasks. When addressing missing data, we employ 2 primary methods: imputation and data removal. Imputation involves substituting reasonable estimates for missing data and is most effective when the incidence of missing data is minimal. In our dataset, missing values for categorical features were imputed with 0, and the median value was used for continuous features. Categorical features were transformed using one-hot encoding methods.^[33]^

### 2.4. Processing

We chose Python Software because it facilitates easy data processing and offers a wide range of libraries available at no cost.^[34]^ We used the open-source Python 3.0 libraries: NumPy, pandas, Matplotlib, PyDot, seaborn, TensorFlow, Keras, and scikit-learn. We used to classification and regression tree (CART) algorithm than can be used for both classification and regression tasks.^[35]^ The dataset was randomly divided into training group (n = 80) and test group (n = 40). We typically adhere to the conventional 70:30 training-to-test set ratio. However, due to the limited volume of data at our disposal, we opted to modify this ratio to ensure a more robust evaluation of the test set performance. This adjustment allows for a more comprehensive assessment under constrained data conditions. The input variables were general characteristics. We utilized the scikit-learn library (GridSearchCV) for hyperparameter optimization. The output variable was OS within 3-months (Alive or Death). To evaluate the tree models, measures such as accuracy, sensitivity, specificity, the receiver operating characteristic (ROC) curve, and the area under the curve (AUC) were calculated, along with their respective 95% confidence intervals (95% Confidence interval). After training the tree models, the important features and permutation features were confirmed. Permutation feature importance is a model inspection technique that can be applied to any fitted estimator when the data is in a tabular format.^[36]^

## 3. Results

### 3.1. Data description

The cohort study included 120 patients with NSCLC, focusing on their age at the time of GKRS treatment. KPS scores and recursive partitioning analysis classes were evaluated by physicians. Pathologic diagnoses, receiving chemotherapy, and pretreatment records were obtained from EMR. The number of lesions, tumor volume, the number of fractions, and prescription doses were documented in the LGP. Detailed data descriptions are summarized in Table 2. Within 3 months following GKRS, 12 patients passed away.

**Table 2:** Summary of histopathological characteristics categorized for machine learning algorithms.

Characteristics	Description	Categorical data for machine learning algorithm	Type
Age	Age at time of treated GKRS	No categorical	Discrete
Sex	Biological sex	0 = Male; 1 = Female	Numeric
KPS score	KPS score runs from 0 to 100. Three physicians allow to evaluate the patient ability to receive GKS for BM	One-hot encoding	Numeric
RPA class	RPA class runs from 1 to 3 Three physicians allow to separate 3 different class	One-hot encoding	Numeric
Pathologic diagnosis	The cell types of the NSCLC cancer	One-hot encoding	Numeric
Pretreatment	Before GKRS, treated the surgery or radiotherapy	One-hot encoding	Numeric
Fixation Methods	Head fixation methods	0 = Frame; 1 = Mask	Numeric
Receiving chemotherapy	This describes if chemotherapy was performed before GKRS	0 = No chemotherapy performed; 1 = Chemotherapy performed	Numeric
The number of lesions	This divided the 3 groups from the number of lesions	One-hot encoding	Numeric
The number of tumor volumes	This divided the 3 groups from the number of volumes	One-hot encoding	Numeric
The number of fractions	Large volume lesions treated hypofractionated GKRS, whereas other lesions treated single session GKRS	0 = Single-session; 1 = hypofractionated	Numeric
The number of prescription dose (50% margin)	GKRS prescription dose from RTOG 90–05 for single-session and linear quadratic model for hypofractionated.	One-hot encoding	Numeric
Overall survival (mo)	The time form the starting of GKRS until the last follow-up time or death	O = Alive, 1 = Dead	Discrete

GKRSBM = brain metastases, NSCLC = non-small cell lung cancer, RTOG = Radiation Therapy Oncology Group.

### 3.2. Evaluation of tree models

**Figure 2.**
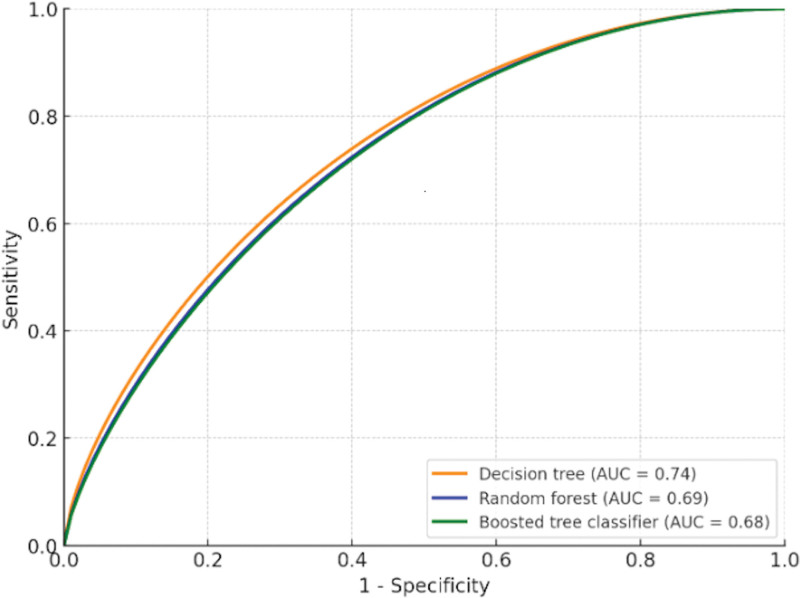
Receiver operating characteristic curves (ROC) and corresponding area under the curve (AUC) values for each model. Predictive performance of machine learning algorithms for overall survival.

The decision tree model achieved the highest accuracy at 77.5%, outperforming the other models, whereas the boosted tree classifier exhibited lower accuracy at 70.00%. Analysis of the decision tree visualized data revealed that patients aged (>71 years old) with tumor volume (>10 cc) were increased risk of mortality within 3-months. The decision tree had the highest sensitivity 80.00% compared to other tree models as detailed in Table 3. The discrimination ability of tree models, as represented by ROC curves and AUC, is shown in Figure 2.

**Table 3:** Predictive outcomes for overall survival using machine learning algorithms.

Algorithms	Accuracy (%)	Sensitivity (%)	Specificity (%)
Decision tree	77.50	90.00	73.33
Random forest	72.50	80.00	70.00
Boosted tree classifier	70.00	80.00	66.66

### 3.3. Evaluation of feature variables

The analysis of important features revealed that age, receiving chemotherapy, and pretreatment were consistently significant features across the tree models. While permutation features identified various prominent features unique to each model, age consistently emerged as an important feature in all models, as detailed in Table 4.

**Table 4:** Assessment of significant variables in machine learning algorithms.

	Important features	Permutation features
Decision tree	Age, receiving chemotherapy, pretreatment	Volume (>10 cc), age, KPS score_90, Number of lesions,
Random forest	Age, receiving chemotherapy, pretreatment	Volume (>10 cc), age, prescription dose (>20Gy), RPA_2
Boosted tree classifier	Age, receiving chemotherapy, pretreatment	Volume (>10 cc), Age, RPA_1, KPS_90

KPS = Karnofsky Performance Score, RPA = recursive partitioning analysis.

## 4. Discussion

This study was designed to investigate the prediction of OS within 3-months after GKRS for NSCLC patients. The main findings of this study are as follows: Decision tree showed higher accuracy compared to others. Important features are age, receiving chemotherapy, and pretreatment. Although permutation features showed different, tumor volume (>10 cc) and age are key input variables.

Graded Prognostic Assessment (GPA) Scores offer clinicians a validated prognostic tool that categorizes patients based on various factors, including age, KPS, and the number of brain metastases. Studies have shown the utility of GPA Scores in forecasting patient outcomes, informing treatment decisions, and stratifying patients in clinical trials.^[37,38]^ Our study suggests that integrating ML with established prognostic tools like the GPA could potentially enhance the precision of patient outcome predictions and personalize treatment strategies. Previous studies have investigated the treatment outcome using ML algorithms for BM patients.^[39–41]^ Vazhenin et al, showed that the accuracy 0.76 for predicting OS for BM patients after GKRS using regression model.^[39]^This approach was shown that significant feature was the largest volume of the lesion by the date of GKRS. Peng et al, investigated that the a radiomics-based prediction model to the problem of diagnosing treatment effect after radiosurgery.^[41]^ The revealed that an optimized IsoSVM classifier based on top-ranked radiomic features had sensitivity and specificity of 65.38% and 86.7%, respectively, with an AUC of 0.81 Improvement in the ability to predict outcomes from the effect for palliative treatment for BM has key practical implications for patients and physicians. In this study, the decision tree showed the accuracy 77.5% in predicting OS within 3-months for BM patients with NSCLC. The results showed slightly improved accuracy compared to other studies. The CART algorithm constructs binary trees with a Gini index as the impurity measure.^[42,43]^ The CART algorithm requires minimal data preparation and allows the for the model accountability to be verified. It can be prone to overfitting or underfitting depending on the tree depth. Random forest and boosted decision trees help minimize these issues. Although decision tree has known more overfitted compared to other algorithms, if decision tree has highly accuracy models, is valuable to considering the clinical fields in each hospital.

Input variables (age, receiving chemotherapy, and pretreatment) had important features on ML to predict OS within 3-months for BM patients with NSCLC. The age of NSCLC patients was found to play a key role model ability. The role of chemotherapy status in the treatment of BM has been reviewed critically die to the failure of most drugs to cross the intact blood-brain barrier.^[44,45]^ Some studies suggests that BM respond to chemotherapy at a rate similar to that seen with the primary tumor and systemic disease.^[46]^ The pretreatment such as ommaya reservoir^[47]^ and WBRT^[48]^ before GKRS could be affected to boosted short OS. Interestingly, permutation features showed different aspects of input variables. Among these, tumor volume size (>10 cc) and age are key factors for predicting OS within 3-months. The volume size (>10 cc) of BM is a relatively contraindication because of radiation toxicity.^[30]^ The Leksell Gamma Knife ICON can treat large BM using fractionated methods with mask-based fixation. However, large BM (>14 cc) compared with smaller tumors, showed no significant differences in OS.^[10]^ The average OS after diagnosis of BM was 18.9 months in patients aged under 60 years and 14.7 months in those aged 60 years or older.^[49]^ We found that older age groups (over 71 years) showed shorter OS in visualized tree models. For elderly patients with large BM, it is considered preferable to treat according to the patient condition rather than overtreatment GKRS.

In this study, there are some limitations. First, the number of patients with NSCLC was relatively small and all data corrected at Chungbuk National University Hospital, thus our model could be possibility for overfitting to our hospital and need to participate the multicenter data in the future studies. Second, the chemotherapy defined to only binary data, not considering to mutation, chemotypes, and so on. Third, this study showed a cross-section of brain tumor development, and we should perform a longitudinal observation of the evolution of NSCLC patients.

## 5. Conclusions

In summary, we investigated to predict OS within 3-months for BM in NSCLC patients. This study show that decision tree algorithm reveals highly accuracy compared to other models. The important features showed that age, receiving chemotherapy, and pretreatment. The permutation features showed commonly showed volume size (>10 cc) and age. We found key factors for predicting OS within 3-months.

## Acknowledgments

This work was supported by grants from the National Research Foundation of Korea (NRF2023R1A2C1008079).

## Author contributions

**Conceptualization:** Young Seok Park.

**Data curation:** Hyeong Cheol Moon, Byung Jun Min, Young Seok Park.

**Formal analysis:** Hyeong Cheol Moon, Byung Jun Min.

**Investigation:** Byung Jun Min.

**Resources:** Byung Jun Min, Young Seok Park.

**Software:** Hyeong Cheol Moon, Byung Jun Min.

**Supervision:** Hyeong Cheol Moon.

**Validation:** Hyeong Cheol Moon, Byung Jun Min.

**Visualization:** Young Seok Park.

**Writing – original draft:** Hyeong Cheol Moon.

**Writing – review & editing:** Young Seok Park.
